# From industrial to digital citizenship: rethinking social rights in cyberspace

**DOI:** 10.1007/s11186-022-09480-6

**Published:** 2022-06-03

**Authors:** Federico Tomasello

**Affiliations:** Robert Schumann Centre for Advanced Studies, European University Institute, Villa Schifanoia, room VL027, Via Boccaccio 121, 50133 Florence, Italy

**Keywords:** Basic Income, Digital citizenship rights, Digital, platform, and surveillance capitalism, Industrial and digital technologies, Industrial citizenship, Social rights and the welfare state

## Abstract

Growing social inequalities represent a major concern associated with the Digital Revolution. The article tackles this issue by exploring how welfare regulations and redistribution policies can be rethought in the age of digital capitalism. It focuses on the history and enduring crisis of social citizenship rights in their connection with technological changes, in order to draw a comparison between the industrial and the digital scenario. The first section addresses the link between the Industrial Revolution and the genesis of social rights. It describes the latter as a legal ‘machine’ designed to offset the imbalances produced by the technological movement of industrialization. The second and third sections introduce the notion of ‘industrial citizenship’ to describe the architecture of social rights in mature industrial societies and to contend that European systems of welfare are still largely modeled on an industrial standard. The fourth part investigates the impact of the Digital Revolution on this model of social citizenship. It identifies debates on basic income as a major trajectory for redesigning welfare regulations in a post-industrial era, and the digital user as a crucial emerging subject of rights. The final part explores how digital users could be entitled to social rights as data suppliers. To this end, it introduces the idea of ‘digital-social rights’ resulting from the incorporation of welfare and redistribution principles into emerging digital rights. Hence, it proposes a legal-political framework for the redistribution of the revenues generated by data in the form of a ‘digital basic income’ for citizens of cyberspace.

As happened in the wake of the Industrial Revolution, technological transformation today appears to be a driving force for new social inequalities. The contemporary data-driven digital economy fosters growing imbalances in wealth and income distribution by facilitating the control of private monopolies over digital infrastructures. The data produced by the online activities of billions of digital users have become extremely valuable assets, but the resulting revenues appear to be increasingly concentrated owing to the disruptive technologies of ‘extraction’ and data monetization developed by giant tech companies. The prevailing standards of data ownership and management resemble vertical silos cornered by major commercial platforms, which bring 19th -century industrial trusts to mind (Petit 2020). Leading scholars in digital studies have stressed how this model of ‘platform’ or ‘surveillance’ capitalism traces and commodifies all aspects of life, annihilates privacy, and threatens democracy, human autonomy, and freedom (Lyon et al. 2012; Boyd & Crawford 2012; Morozov 2013; Stiegler 2016; Srnicek 2017; Ruha 2019; Couldry & Mejias 2019; Zuboff 2019). Yet, far less attention has been paid to possible redistribution measures which could be adopted to mitigate the new social imbalances produced by platformization and datafication processes. This article addresses the relation between digital technologies and inequality by investigating how social rights and welfare policies could be rethought in the age of digital capitalism and implemented into the current architectures of cyberspace. The guiding questions are: on what normative and legal basis could digital users, as data producers and suppliers, be entitled to social rights and redistribution policies? How could part of the wealth generated by digital data be redistributed to digital users based on the establishment of new social rights belonging to the digital sphere? To unpack these questions, the article first focuses on the history and enduring crisis of traditional welfare regulations to assess how they can be redesigned in the changing technological scenario of our age. Then, it explores the way in which emerging digital rights can support the deployment of new redistribution policies within cyberspace, understood as an additional sphere of citizenship rights brought into being by digital transformation.

This approach is inspired by the Marshallian conceptualization of modern citizenship as the progressive historical sedimentation of civil, political, and social rights (Marshall 1992). Even if it has been rightly criticized for its teleological approach (Bottomore 1992; Turner & Hamilton 1994; Bulmer & Rees 1996), this canonical definition provides a useful platform to explore possible connections between traditional *social rights* and emerging *digital rights*. Specifically, Marshall has described how the advent of social citizenship has also reshaped civil and political rights through the incorporation of welfare and redistribution principles––e.g., through the introduction of the right to legal aid in courts and of remuneration for political representatives to enable workers to hold these positions. Accordingly, this article investigates how social citizenship principles can be incorporated into a new generation of rights belonging to the digital sphere―such as access, openness, net-neutrality, encryption, and, above all, the right to protect and control personal data. This means rethinking digital rights beyond the sole issue of privacy to which they are usually confined and––at the same time––reframing social rights so as to encompass the key role of cyberspace in creating economic value as well as new inequalities.

Even though the global digital economy currently has the US and China as its main powerhouses, this article focuses on the European case for several reasons that can be elucidated by describing the paper’s structure. The *first part* considers the aftermath of the Industrial Revolution in order to retrace the genesis of social rights as a legal technology aimed at mitigating the inequalities engendered by the advent of the industrial age. The *second and third parts* draw upon the notion of ‘industrial citizenship’ to frame this link between industrialization and welfare policies and to describe the architecture of social rights in mature industrial societies based on mass-scale production, a vocation to full employment, and the ‘citizen-worker’ as the quintessential subject of rights. Such a reading of social welfare history focuses on Europe because it is where this institution originated and developed a specific connection with citizenship as a legal status that entails universal and constitutionally recognized social rights (Rueschemeyer & Skocpol 1996; Polanyi 2001; Castel 2003; Castles et al. 2010). Indeed, social citizenship has come to be regarded as the defining element of a ‘European model’ of society; a model which nonetheless has entered into crisis over the last three decades, owing to several factors, including technological transformation, that is the focus of this article.

The *fourth part* discusses the crisis of European welfare systems in the changing technological scenario of our age. It maintains the need for a new political anthropology of social citizenship going beyond the figure of the citizen-worker that has characterized the industrial scenario. Hence, it identifies the digital user as a crucial emerging subject of rights, and debates on basic income as a major avenue for redesigning welfare regulations in a post-industrial coontext. The *fifth part* explores how digital users could be entitled to social rights as data suppliers. It proposes a legal-political framework for the redistribution of the revenues generated by digital users’ data in favor of their producers and suppliers. Such a framework is based on the analysis of potential developments of the right to protect and control personal data, as established by the EU General Data Protection Regulation (2018). Consequently, the article’s main focus remains the EU, owing to its growing effort to become a ‘regulatory superpower’ in digital governance. As highlighted in the *conclusion*, this regulatory effort also entails the proposal for a ‘Tech’ or ‘Web’ Tax. This article aims to outline a rights-based approach to such a topic by connecting it to the institution of citizenship as the main carrier of rights in Western societies and by framing it within an intellectual-historical analysis of the mutual relation between technological changes and citizenship rights.

## Citizenship and technology: on the genesis of social rights in Europe

Since the French Revolution, the institution of citizenship has developed into a major carrier of rights and a powerful driving force for *equality* among members of the same nation. The 1795 *Declaration of the Rights of Man and of the Citizen* stated that ‘the law is the same for all’ (art. 3) and that ‘each citizen has a legal right to participate directly or indirectly in the formation of the law’ (art. 20). These principles of civil equality and of equal political rights established the legally homogeneous national citizenry and brought about a consistent definition of modern citizenship (Brubaker 1989). Though limited to free men, this institution aimed to define a legal-political sphere of general human equality despite all the differences shaping the other spheres of human existence (Tilly 1995; Hammersley 2015). In parallel to the spread of these legal-political principles throughout Europe, the First Industrial Revolution began to transform post-revolutionary societies and fostered the rise of new imbalances in the social realm. Besides citizens’ civil and political equality, the Revolutionary Declarations affirmed private property as an ‘inviolable and sacred right of man:’ the Industrial Revolution turned out to be a powerful driving force of *inequality* in such a domain. Indeed, the advent of an industrial regime of production and commerce created the conditions for almost limitless inequalities of wealth. Thus, the citizen rights established by political revolutions made it possible to overcome old structures based on status, at the same time as new forms of human hierarchy were being created by industrialization in the social sphere. In this regard, the social consequences of the Industrial Revolution conflicted with modern citizenship as a system of equality and it is in light of such a tension that I propose we understand the European genesis of *social rights*.

Alexis de Tocqueville stressed this contradiction by stating: ‘if ever a permanent inequality of conditions and aristocracy again penetrate into the world, it may be predicted that this [industrialization] is the channel by which they will enter.’^1^ His writings on this matter reveal that the political and social theorists of the early 19th century perceived the main feature of the rising industrial regime as an antinomy: the simultaneous and antithetical growth of unprecedented wealth and of new forms of poverty and harsh deprivation. When visiting Manchester, Tocqueville (1958: 107–8) wrote:

Look up and all around this place you will see the huge palaces of industry. You will hear the furnaces, the whistle of steam. […] here is the slave, there the master; there the wealth of some, here the poverty of most […]. Here the weakness of the individual seems more feeble and helpless than even in the middle of a wilderness; […] From this foul drain the greatest stream of human industry flows out to fertilize the whole world. From this filthy sewer pure gold flows. Here humanity attains its most complete development and its most brutish; here civilization works its miracles, and civilized man is turned back almost into a savage.

These words reveal two crucial aspects of the contradiction that emerged between the political movement of egalitarian citizenship and the new inequalities triggered by the technological movement of industrialization. First, to describe such inequalities Tocqueville draws upon the opposition between ‘slave’ and ‘master’, ‘civilized man’ and the ‘savage.’ These dichotomies point to the fact that the first representations of the subaltern subjects brought into being by the Industrial Revolution were built upon a reference to colonized populations, used to describe a sort of anthropological or even racial difference (Virdee 2019). Industrialization fostered the mass migration toward manufacturing cities of a growing number of working poor coming from backward rural areas. These new ‘dangerous classes’ were perceived as somehow foreign, as ‘external’ to the social body of the nation, and were described by comparing them to the ‘savages’ of the colonies or, especially in France, to a ‘new barbaric invasion’ (Chevalier 1973; author forthcoming). This ‘racialization’ of the emerging European proletariat (Robinson 1983) served to define a condition of radical otherness and foreignness with respect to the national citizenry established by modern civil and political rights. The growth of this ‘industrial class’ of propertyless citizens and its state of deprivation and social exclusion embodied a stunning contradiction with respect to the egalitarian principles affirmed by political revolutions. Hence, the inclusion of these subjects within a condition of essentially equal citizenship progressively became the main challenge for 19th -century European societies. Social rights gradually emerged as the legal technology to address this issue and to include the social dimension within the institution of citizenship by furnishing the fabric of industrial societies with legal devices to reduce the gap between formal equality and substantive inequality among citizens.^2^ In such a way, the tension between the inclusive rationale of modern citizenship and the effects of social exclusion produced by industrialization nurtured the rise of modern welfare principles (Rueschemeyer & Skocpol 1996; Kuhnle & Sander 2010).^3^ Accordingly, the web of social rights that were to inform modern welfare states ‘was designed as a machine to correct the imbalances caused by industrial progress’ (Supiot 2017: 2).

This understanding of the genesis of social citizenship points to the second aspect emerging from Tocqueville’s words: the internal ambivalence of the Industrial Revolution in fostering the parallel and exponential increase of prosperity and deprivation, wealth and inequality, emancipation and subjugation. Industrialization required economic resources and therefore those who benefited the most from the first industrial developments were capital providers―in a legal and political context shaped by economic liberalism and the primacy of private property and entrepreneurship. This raises the question of the very link between inequality and technological innovations, which are mostly driven by profit opportunities and investments resulting from capital availability and investors’ preferences (Bénabou 2005; Saint-Paul 2008). Neither technology nor the disruptions that come with it are mere exogenous forces stemming from advances in science and driving industrial development from the outside. Instead, they depend on the contingent state of industry and the labor market, and require favorable socio-economic conditions in order to become embedded in the industrial fabric and to transform it (Romer 1990; Aghion & Howitt 1998; Acemoglu 2003). Although the technical-scientific conditions had long since matured, the defining technology of the First Industrial Revolution―the steam engine―only emerged after a series of previous transformations that enabled it to radically transform manufacturing processes. The new regime of land property determined by the enclosure of common fields; the resulting availability of capital and a migrant workforce; the rise of big manufactures, whereby hand-production was enhanced by the division of labor and new instruments; the expansion of markets; and other concomitant processes created the conditions for the steam machine to be adopted and to have a disruptive impact on the textile industry (Dobb 1963; Horn et al. 2010). In that context, the main restraint on the further development of production resided in the limits of the physical energy of the human workforce and steam energy made it possible to overcome such a limit (Habakkuk 1962; Zmolek 2013). Previous capital accumulation made this technological shift possible: so-called ‘primitive accumulation’ preceded industrialization and served as the pivotal driving factor for it, according to its own logic of development (Marx 1867).

Technology is aimed at providing solutions, but the hierarchy of problems to be solved depends on unequal structures of opportunity and power. Steam machines were the solution for those who were committed to a manufacturing system marked by an energy deficit and had resources to invest in such technology, by which the physical energy of men and animals was replaced with that of industrial machines. The mechanization of production resulted in an extensive division of labor that made it possible to replace craftsmen with low-skilled workers, entrusted with easier and more repetitive tasks. Not least owing to the revolutionary abolition of all intermediate bodies―including guilds with their ancient forms of social security―these industrial workers experienced a new condition of social exclusion and deprivation that, from the 1830s, came to be referred to through neologisms such as ‘pauperism,’ ‘proletariat,’ and ‘social question’ (Ewald 1986; Castel 2003). Their formal condition as equal citizens was deeply affected by the inegalitarian dynamics triggered by the capitalistic use of industrial technologies. Thus, we can recognize an emerging tension between citizenship as a principle of equality and the inegalitarian dynamics that technological changes tend to produce in society. And we can look at the ‘invention’ of social citizenship rights as a legal-political technology designed to mitigate the dynamics of inequality fostered by technological developments.

The latter tend to be driven by investors and shaped by asymmetric assets in terms of incentives, resources, and knowledge. This explains their connection with rising socio-economic inequalities, as well as the fact that technological change—at least in its early stages―often proves to be a key driver for both aggregate economic growth and rising imbalances in wealth and income (Burnett 2009). These assumptions apply to the mechanical turn of the First Industrial Revolution, but can also offer a key to interpret the new inequalities emerging in our age from the economic disruption caused by ICT and digital technologies. Indeed, the growth of inequality represents a major public concern associated with both of these historical phenomena (Brynjolfsson & McAfee 2014). Since the last quarter of the 20th century, digitalization processes have allowed artificial intelligence to replace some intellectual performances of the human brain in an economy increasingly based on information and communication (Bodei 2019). These labor-saving technologies have increasingly affected ‘middle class,’ ‘middle-skill’ and white collar jobs and supported the rise of a ‘gig economy’ decreasing the cost of labor in favor of corporate profits (Friedman 2014; Rodrik 2015; Frey 2019). Furthermore, over the last two decades, a data-driven economy has emerged and developed into a winner-takes-all model that facilitates private monopolies’ control over digital infrastructures (Petit 2020). To date, these trends have produced new inequalities resulting from ‘technological unemployment,’ ‘job polarization,’ and the concentration of revenues in the hands of those who have enough capital to acquire labor-saving technologies and/or control tech infrastructures (Schwab 2016, Frey & Osborne 2017; ILO 2020; MIT 2020). Thus, technological change appears to play a major role among those factors fostering the growing inequalities of our age (Piketty 2013). Of course, these inequality dynamics have a profoundly different nature compared to the 19th -century ‘social question,’ and therefore they should be addressed through different legal tools and policy responses. To assess these differences, the next two sections will draw upon the notion of ‘industrial citizenship’ to describe the architecture of social rights in the post-WWII European scenario, marked by the full establishment of welfare states. This analytical framework will then be used, in the last two sections, to assess the effects of digital transformation on existing systems of social citizenship.

## Industrial citizenship:’ social rights as industrial rights

Industrialization has been much more than a technological and economic process. It has developed into what we might call―in M. Weber’s words―a ‘world image’ (*Weltbild*): a systematic understanding of our position in the world that defines the horizon of our expectations, and the tools needed to achieve them (Weber 1974, 1978; Kalberg 2004). It has been an epoch-making movement informing the idea of the future as progress. It has defined an era of Western civilization: the Industrial Age. Major sociologists like Dahrendorf have agreed in describing the post-WWII landscape in terms of ‘industrial societies’ and in assuming that the ‘quintessence of reality’ should be sought within the industrial realm (Dahrendorf 2004: 184; 1959), which is to say within the big industries shaped by the *Second Industrial Revolution*. The latter occurred from the late 19th century onward through the introduction of new energies, such as hydroelectricity and oil derivatives, new materials, such as thermoplastics, and new inventions, such as internal combustion engines. Electrification fostered mass-scale production and the ‘scientific management’ first epitomized by F.W. Taylor’s *Principles* and by the assembly line of Ford’s Model-T factory. This was not just a production regime. Rather, it forged an entire social order, since it required and promoted the growth of a ‘mass consumption’ society, which was supported by wage increases, social insurances and a form of public demand management known as Keynesianism (Beveridge 1942; Crouch 2009). In this way, industrialization has reshaped the modern edifice of citizenship and growing social rights have offered a crucial legal platform for this effective entanglement between industry and society (Titmuss 1963; Esping-Andersen 1990). To designate the resulting model of citizenship, I will draw upon the notion of *industrial citizenship*, coined by T.H. Marshall (see the next section), and reframe it to describe the link between industrial technologies and social rights.

Schools, hospitals, and factories are the institutions that epitomize both the bureaucratic logic of concentration, centralization, and standardization characterizing industrial societies and the three main social rights of citizenship―access to education, health care services, and the right to work. These rights supported the growth of a secure working class able to enjoy full inclusion in citizenship guarantees and to become confident mass consumers within a mass-production economy. This entanglement between social rights and industrial production ensured the stability of the social order before the entropy and ‘creative destruction’ of the capitalistic economy. Accordingly, the welfare state was designed as a ‘machine’ aimed at balancing the effects of industrial disruption by redistributing, on a national scale, part of the wealth produced by industries in the form of social services. In this sense, social rights can be understood as *industrial rights*. And we can use the notion of industrial citizenship to designate the common systemic rationale binding together the unfolding of social rights in the legal-political sphere and the development of industrial societies.

This bond is emphasized by Marshall (1992: 37) when analyzing the genesis of the social right to education ‘as a means of providing capitalist employers with more valuable workers and higher education as an instrument to increase the power of the nation to compete with its industrial rivals.’ He furthermore stresses that, after the first laws on child and women’s labor, ‘the factory code had become one of the pillars in the edifice of social rights’ (ibid.: 15). This was a legal but also material ‘pillar’, since national industrial production was, via taxation, the main financial source for the welfare state, and therefore the pivotal condition for its very existence. Thus, industrialization appears to be the cradle of social citizenship rights. And such an intertwined development of industrial technologies and social rights suggests that technological change can be understood as a crucial factor in the 20th -century evolution of social citizenship―in addition to the social and political forces usually stressed by citizenship studies. As we have seen above, this does not mean looking at technological innovation as an autonomous force; rather, it implies taking into account the way in which technology has shaped essential notions, strategies and institutions that are inherent in a citizenship regime marked by the advance of social rights.

Industrial technologies have forged the conception of *labor* that has inspired European systems of social citizenship, that is a specific historical figure of labor: male adult paid employment (Scott 1988; Moulier Boutang 1998). This kind of labor stands as the cornerstone for both the capitalistic production of value and the full deployment of social rights, whose blueprint and driving principle must be sought within the industrial fabric and identified in the right to work. This is an industrial conception of labor that also entails a division, based on a gender hierarchy, between paid ‘productive’ labor and unpaid ‘reproductive’ labor (Laslett & Brenner 1989; Pateman 1989; Glenn 2002; Duffy 2007). Such an implicit yet powerful division has played a crucial role in designing welfare regulations in mature industrial societies (see the next section). Industrial technologies of mass production have also defined the horizon of *full employment*, which is a constitutive aspect of the idea of industrial citizenship. A constant process of industrialization has embodied the precondition for full employment, which has been actively pursued by governments in order to fulfil *the right and duty to work* for all citizens as the pillar of substantive democratic citizenship (Montgomery 1993; Pixley 1993).

As a result, we can regard the edifice of European social citizenship as one built on industrialist foundations and on an industrial representation of society. And we can think of ‘industrial citizenship’ as a legal-political architecture for manufacturing the social according to the centrality of industrial production and labor. My argument is that contemporary welfare regulations in Europe are still largely shaped by these principles of industrial citizenship designed to counteract the inequalities produced by industrial technologies (Supiot 2017). And that this connection with the industrial regime can provide a promising key to interpret the enduring crisis of such regulations in the changing technological context that, over the last four decades, has profoundly reshaped European industries and labor markets, inequality structures and even the very idea of labor. By transforming these foundations of welfare systems, ICT and digitalization processes have undermined the traditional architectures of social citizenship. Before addressing this impact and the resulting crisis, it is useful to further reframe the idea of industrial citizenship as a system of rights and duties.

## The citizen-worker

Even if T.H. Marshall’s influential treatise on *Citizenship and Social Class* (1950) rests on a ‘liberal’ understanding of citizenship as a bundle of rights, it also expresses a ‘republican’ perspective when discussing the *duties* of citizenship. In his view, the latter appear to be only vaguely ‘included in the general obligation to live the life of a good citizen’ and ‘to promote the welfare’ of the national community, which nonetheless is too ‘large,’ ‘remote,’ and ‘unreal’ to command a ‘keen sentiment of loyalty’ and substantial ‘acts of will’ (Marshall 1992: 45–47). Hence the need for ‘more limited loyalties,’ which Marshall envisages as more likely to emerge in the closer dimension of industrial workplaces and which he designates as forms of ‘industrial citizenship.’ ‘In times of full employment,’ Marshall (1992: 46) argues, the ‘general duty to work’ should be rethought so as ‘to revive the sense of the personal obligation to work in a new form in which it is attached to the status of citizenship’ and which implies the duty ‘to put one’s heart into one’s job and to work hard.’ The Marshallian notion of ‘industrial citizenship’ designates a form of belonging and civic engagement rooted in the workplace as a fundamental space for nurturing civic virtues and a public-spirited citizen-worker.^4^ In such a way, the classical-republican theme of the civic virtues of citizenship is reformulated as a matter which can be found within industrial workplaces, in a sort of professional ethic that frames labor as a social duty and civic virtue. This is a work ethic that also marks most post-WWII European constitutions―e.g., the Italian one, whose first article states that ‘Italy is a democratic Republic founded on labor.’

In this idea of industrial citizenship, it is through work that an individual becomes a full citizen and expresses himself as such; participation in the public life of the political community is tied to participation in the labor market; and labor ‘discipline’ is considered to crucially underpin the social order (Del Re 1994; Foucault 2012). Thus, the figure of the ‘good citizen’ tends to reflect that of the decent industrial worker who contributes to national welfare through his daily hard work. This conception also reveals a ‘sacrificial’ approach to work, understood as a moral obligation, social duty, and public value regardless of the harsh and petty material forms it often takes–e.g., in the assembly lines of big factories. In this sense, we can regard 20th -century industrial societies as being based on an *industrial social contract*, which exchanges social security and a basic standard of living for subjection to the discipline of a repetitive and alienating work producing the nation’s wealth. Social citizenship has embodied the legal technology making such an exchange possible through the redistribution of industrial revenues according to this industrial social contract. The latter has been conceived of within a strictly national frame and in such a way as to ultimately make the growth of the national working class part of the development of the state. This has defined the *boundaries* of industrial citizenship and its exclusionary features, which have been radically challenged by globalization processes fostering massive translational migrations of the workforce (Kochenov 2019).

Although the notion of industrial citizenship has been coined by Marshall to frame labor as a virtue and duty of citizenship, in my view it has the potential to also encompass the entire web of social rights in mature industrial societies. If, following Marshall, we understand industrial citizenship as a form of civic engagement based on labor, we should understand the *right to work* as the pillar of all social rights associated with industrial citizenship. Indeed, post-WWII European constitutions define labor as a fundamental right and duty of all citizens, to be promoted by the state in order to achieve substantive democratic citizenship (Taylor 2000).^5^ In this context, the very idea of social security primarily designates employment security, and the structure of the welfare benefits embodying social citizenship depend, to different degrees, on work conditions. Most social rights are conceived of as an insurance for workers who are unable to participate in the labor market: social insurance for sickness, injuries, and disabilities, pensions as a social right upon retirement, insurance for unemployment, active labor market policies, family benefits, etc. The elderly, the unemployed, and the poor living on welfare are regarded as a workforce that has been, permanently or temporarily, excluded from the job market owing to objective circumstances: they are entitled to social rights because of this condition (Beveridge 1942). Though formally universalistic, the social rights of industrial citizenship therefore reveal an implicitly conditional character, since they are closely connected to employment conditions (Castles et al. 2010). Thus, we can understand the entire edifice of European social citizenship as being built around a specific subject of rights: the dyadic figure of the *citizen-worker* that is inherent in mature industrial societies (Glenn 2002; Mezzadra & Nielsen 2013: 250). In these societies, labor and citizenship have become overlapping institutional spheres and their nexus has shaped the whole architecture of social rights (Kvapilova 1995).

This has been a specifically European architecture, marked by the constitutionalization of social rights and the role played by trade unions as major institutional actors. Hence, this idea of industrial citizenship would require a different framing for the US, where it has been brokered mainly by individual employers, as well as for those East Asian states that have developed forms of ‘welfare productivism’ or ‘developmental welfare’ (Tang 2000; Hwang 2012). Instead, the model I have outlined reflects in particular so-called ‘conservative’ systems of welfare, which are typical of countries like Germany, France, and Italy. These mainly depend on workers’ statuses or professions and chiefly apply to regular employees in traditional economic sectors (Esping-Andersen 1990; Stryker & Eliason 2003; Palier 2010): as such, they display a clear link with the industrial paradigm and the kind of difficulties in adapting to contemporary technological transformations that I will addresses in the next section. We can therefore describe European systems of welfare as based on an implicit industrial anthropology identifying labor with paid employment and the figure of the citizen with that of the worker as the quintessential subject of rights. Along with other factors, digital transformation has clearly had a momentous and disruptive impact on this close nexus between labor and citizenship. In the next section, I will investigate such an impact with the aim of exploring, in the last section, the way in which social citizenship could be rethought in the changing technological scenario of the digital age.

## A ‘post-industrial’ revolution?

The epoch-making technological turn of our present time is often described as the Fourth Industrial Revolution (Schwab 2016), coming after the First and Second revolutions considered in the previous sections and the Third one, resulting from the spread of information and communication technologies and the shift from industrial production to services (Touraine 1971; Bell 1973; Wiener 1989; Castells 1996). Yet, for the purposes of this article it is more interesting to stress how digital transformation has actually accompanied the decline of the Industrial Age in the Western world. Along with other major concomitant factors such as financialization and globalization processes, and the ‘neoliberal’ turn in economic governance, the advent of digital technologies has led to extensive processes of ‘deindustrialization,’ with the result that the present European landscape has increasingly come to be described as ‘post-industrial.’^6^ In this regard, contemporary digitalization processes could be framed as either the Fourth Industrial Revolution or as the ‘first post-industrial revolution.’

This post-industrial understanding of the notion of *Digital Revolution* is based on two central features shaping the present article’s perspective: a radical rescaling of the role of human labor in the value chains of an economy increasingly driven by data––on which I will focus in the following pages––and the creation of the online––or ‘onlife’––digital environment (Floridi 2015). While the Industrial Revolution fostered the rise of the ‘social’ sphere as a new dimension of rights, the paradigm of the Digital Revolution is based on technologies producing cyberspace as a new spatiality of human existence, which requires new epistemological and normative frameworks to incorporate an architecture of rights. Just as industrialization has forged a specific image of our world (Musso 2014), so digitalization is enabling new ways of seeing and understanding the world through ‘algorithmic knowledge’ based on Big Data analysis (Chandler & Fuchs 2019; Amoore 2020). Just as an industrial imaginary inspired the representations of 20th -century societies––and informed welfare regulations accordingly––so IC technologies have fostered a new cybernetic imaginary that shapes the ways in which we think of our social reality (Wiener 1989; Pickering 2010; Supiot 2017). Nevertheless, social citizenship in Europe still appears to be founded on an implicit industrial anthropology and representation of society, and on an image of the main subject of rights forged by the industrial era.

In particular, social security systems are still largely modeled on a work standard that has ceased to be typical. While employment is still considered a major citizen right, duty or even virtue, and still represents the main carrier of inclusion into the institutions of social citizenship, social rights claims on the grounds of lifelong full-time paid employment can no longer be considered the norm (Dore 1996; Standing 2009, 2014; Van Parijs 2017). Digital automation is increasing the number of jobs performed by thinking machines, making ‘technological unemployment’ a relevant feature of the current landscape (Frey 2019). Even if this trend may not be general but only contingent, digitalization is disrupting almost every industry and a major aspect of this disruption nowadays has to do with the declining role of human labor as paid employment (Brynjolfsson & McAfee 2014; Frey & Osborne 2017; Helbing 2019). While work used to be the central factor for value creation in industrial capitalism, the value chains of digital capitalism appear to have become partly independent of labor (Fisher & Fuchs 2015; Frayssé 2015). Giant tech companies seem to be the equivalent of cotton and spinning factories in the aftermath of the First Industrial Revolution, but their business model requires a far lower amount of paid employment. The magnitude of these phenomena is vast enough to call into question the very social contract underlying our labor-based societies and systems of welfare.

The enduring crisis of social citizenship in Europe has to do, first of all, with this decline of work as it was experienced in the mature industrial era; a shift that is turning the horizon of full employment into an illusory goal in several European countries (Coenen & Leisink 1993; WEF 2016). The very nature and meaning of labor have been profoundly challenged by digital transformations, to the point that even the simple dichotomy between employed and unemployed is losing its heuristic value. New social cleavages are growing between ‘citizen-workers’ who are still fully included in welfare regulations and the emerging figures of a contingent and precarious workforce, reproductive and unpaid laborers, gig workers, and underemployed citizens who are––partly and to different degrees––excluded from such regulations (Offe 1992; Parrenas 2001; Cheah 2007; Standing 2011, 2014; Hester 2018; Schor et al. 2020). These trends embody a radical challenge to the architecture of rights I have described as ‘industrial citizenship.’ The latter reveals an increasing difficulty in dealing with the new social imbalances created by the transition to a post-industrial and digital era, which is profoundly eroding the nexus between labor and citizenship that shaped traditional welfare guarantees. In my view, this fracture brings up the need for a new political anthropology of social citizenship capable of overcoming the dyadic figure of the citizen-worker as the quintessential subject of rights.

Against these major transformations, the idea of a *universal basic income* understood as a citizenship right is gaining attention. In the last few decades, it has been endorsed by a growing number of scholars as a means to rethink welfare regulations in a context marked by technological unemployment, work precariousness, and a race toward labor-saving technologies (Standing 2009; Van Parijs 2017; Torry 2019; Huws 2020). Such interest can be found across a vast political spectrum, ranging from critical and radical theorists to neoliberal thinkers who have developed the idea of a negative income tax (NIT) aimed at reducing state regulation.^7^ Even the business community of the Silicon Valley has promoted basic-income pilots (see the Y Combinator program), while proposals for a drastic reduction of the work day and week, aimed at reviving the right to work for all, have also emerged as a different way of facing the socioeconomic decline of labor.^8^ Thus, the complex politics of basic income ideas ultimately reflects broader perspectives concerning the role of labor in our societies and models of citizenship rights and social recognition (Brynjolfsson & McAfee 2014). My aim here is to explore how basic income claims could be framed within the digital sphere as a dimension that has yet to be consistently permeated by citizenship principles and architectures of rights.

In general terms, universal basic income (UBI), a basic income guarantee (BIG), or simply a citizen’s income (CI) is a scheme ensuring that all citizens have enough income to meet a decent standard of living regardless of their position on the job market. In this sense, UBI has the potential to expand the political anthropology of social citizenship beyond the dyadic figure of the citizen-worker that has characterized industrial societies. A basic income guarantee can be understood as a social citizenship right aimed at mitigating changing forms of social risk and economic insecurity as well as shocks and hazards such as the 2008 financial crisis and the current pandemic outbreak. These crises have furthered public interest in UBI by highlighting the inadequacy of established welfare guarantees in dealing with their harmful social effects (Torpey 2020). Yet, reasonable criticisms and doubts persist concerning the feasibility and desirability of citizen’s income schemes. Among them, there are widespread concerns about the means of financing basic income and the possible tensions it might create between working taxpayers and the main UBI beneficiaries, who remain outside, or at the margins, of the job market. To face these criticisms, basic income guarantees should be rethought beyond the established framework of 20th -century welfare regulations, in order to fully encompass the growing economic and societal relevance of the digital sphere. In what follows, I aim to indicate how debates on UBI could embrace the paramount role of cyberspace in creating economic value as well as new social inequalities, and therefore its key importance for any project of wealth redistribution in our age.

In the contemporary world, the Internet has become a core center for the creation of vast amounts of wealth that are increasingly proving to be unequally distributed, not least owing to the declining role of work as gainful employment in a digital economy. A growing part of economic life is taking place in cyberspace, but human labor seems to be fading in this global and immaterial dimension. Most European systems of social citizenship do not easily adapt to this changing technological scenario since they have been designed on a national scale in industrial societies marked by the vocation to full employment and, consequently, have assumed occupation as the main platform to access welfare regulations. In this context, UBI represents a way of thinking about social security that departs from the idea that paid employment is the fundamental condition to create wealth for a national community. This perspective becomes more cogent in the framework of a digital economy whose business model is largely based on the ‘extraction’ of economic value from information provided by digital data (Zuboff 2019). This model deploys forms of ‘growth without labor’ marked by the dissociation between paid employment and the value chains characterizing the digital economy―particularly the Big Data value chain (Faroukhi et al. 2020). Social media and digital platforms epitomize these trends toward an economic paradigm that makes the creation of wealth partly independent from paid work.

In this scenario, the centrality of the citizen-worker has been challenged, blurred, and complemented by new emerging profiles, like that of the *digital user*. As we are spending more and more of our life in cyberspace, this figure has come to embody several subjects of rights: a digital user may be a consumer of goods, a service user, an online platform worker, a data producer or supplier, a media and information consumer, an online learner, an active and public-spirited citizen. Indeed, cyberspace is catalyzing political participation whereas the fundamental site of citizens’ engagement is no longer the workplace (Howard 2006; Papacharissi 2010; Isin & Ruppert 2015; McCosker et al. 2016; Hintz et al. 2019). Accordingly, while traditional social rights have long entered into crisis, a new generation of rights belonging to the digital sphere has emerged―such as the rights to internet access, privacy, the protection, encryption and control of digital data, net neutrality, and digital sovereignty. In the next section, I aim to explore possible interplays between social rights and digital rights in order to assess how social citizenship principles could be incorporated into emerging digital rights, especially with regard to the redistribution of the wealth generated by digital data. My goal is to advance a normative framework for the implementation of a digital form of basic income by framing it as an emerging ‘digital-social’ citizenship right. To this end, I will investigate on what basis a UBI scheme could be developed within the legal-political architectures of the digital sphere understood as an additional dimension of citizenship rights.

## Digital-social rights

The monetization of data is at the core of the business models of digital capitalism. Everything digital users do in cyberspace leaves data traces which are amassed and analyzed through data-matching and data-mining algorithms to construct personal profiles and digital personae. These profiling data are then sold multiple times, generating revenue for different stakeholders and making users’ clicks an exponentially valuable product (Zuboff 2019). The wealth produced by Big Data value chains―e.g. through the online advertisement market―is now in the order of many billions of dollars a year.^9^ Ubiquitous datafication rewards the few who control digital infrastructures and, as a source of inequality, the resulting revenues should be considered crucial for redesigning redistribution policies in the digital age. Besides, users’ data are the raw material needed for the very advancement of digital technologies, in particular those based on artificial intelligence and machine learning, which grow through Big Data analysis (Amoore 2020; Fourcade & Johns 2020). We might posit, then, that the activities of digital users generating data in cyberspace are a pivotal force of value creation in digital capitalism, just as labor was in industrial capitalism. Accordingly, digital users should be considered not only consumers but also *data producers* and *suppliers.* This assumption immediately raises two pivotal questions concerning possible developments in digital citizenship rights. On what normative and legal basis can digital users, as data producers and suppliers, be entitled to social rights and redistribution policies? What digital right offers an effective platform for the inclusion of social citizenship principles within cyberspace?

My argument is that, to tackle these questions, we should primarily focus on the evolution of the right to digital privacy into *the right to protect and control personal data*. This evolution indeed represents a crucial step toward the legal recognition of digital users as data suppliers. The EU General Data Protection Regulation (GDPR)―agreed upon by the European Parliament in 2016 and effective as of 2018―has been a pioneering measure in this direction, which has already been followed by some twenty countries throughout the rest of the world. It has the potential to reshape current architectures of cyberspace by regulating data governance in order to protect digital users as bearers of rights. However, in the GDPR internet users have been defined using the limited and contested term ‘data subjects,’ entitled to rights mainly as consumers, and protected exclusively on the privacy level. How can these data subjects and digital consumers obtain full digital citizenship with equal rights in the domain of data? What rights do digital users have as data producers with respect to the economic value created by their activities in cyberspace? If digital users have the right to exercise full control over the data they supply, this should also apply to the commercial use made of such data; consequently, digital users could claim a *‘digital-social’ right* with regard to the monetization processes of their data. It is on this basis that social welfare regulations could be extended to the digital sphere, in order to redistribute part of the wealth created by datafication.

The value generated through data analysis has a ‘social’ or ‘common’ root that lies in the digital relations of internet users and in the ‘fundamentally social’ character of ‘data production in the digital economy’ (Viljoen 2021: 580). According to Fezer (2017, 2018), data are ‘behavior generated’ and result from users’ interactions as forms of ‘cultural agency.’ The claim that digital users bear permanent rights over the data they produce raises the question of who actually owns the data and of what the nature of that ownership might be (Hummel et al. 2020). Social media and platform capitalism are largely based on contracts that exchange data ownership for a digital service, through which digital users surrender the rights to their own data to platform companies (Gorwa 2019). This legal exchange―which lies at the core of the platform economy―is exclusively ruled by the market and has not yet been subjected to an architecture of rights aimed at protecting platform users as digital citizens. In this respect, digital platforms are reminiscent of labor relations in 19th -century factories, which were only regulated by market forces through private contracts in accordance with the ‘laissez-faire’ economic orthodoxy of the age. This analogy allows for a further development of the comparison between ‘industrial citizenship’ and the digital scenario aimed at assessing how welfare principles could be implemented in data-driven economies and ‘datafied societies’ (Hintz et al. 2019).

As we saw above, social citizenship originated from the recognition of rights within the industrial domain of labor. Social rights have progressively penetrated and reshaped this industrial sphere through egalitarian citizenship principles aimed at protecting citizen-workers through social security measures. Accordingly, Esping-Andersen (1990: 21) has described social rights as a ‘decommodification of the status of individuals vis‐à‐vis the market,’ since welfare regulations have transferred the allocation of certain goods and services from market determination to political determination (Stephens 2010). Thus, European social citizenship has incorporated a logic of citizen rights in addition to market rules in regulating certain social relations, particularly in the domain of industrial labor. As a result, welfare states have functioned as systems of redistribution of the wealth generated by industry on a national scale. In our digital age, these principles of social citizenship should be rethought in the realm of cyberspace in order to properly frame digital users as citizens and subjects of rights also with regard to their role as data producers/suppliers. In such a way, the emerging notion of digital citizenship can be connected to the epistemological framework of social welfare studies, so as to encompass principles of redistribution that are inherent in modern social rights. This connection makes it possible to introduce the notion of *digital-social citizenship* and to develop it according to the assumption that citizens have rights with regard to the economic value created by the data they supply―i.e., to their monetization processes.^10^

Such a rights-based approach to data monetization calls into play the way in which emerging digital rights can incorporate values of social equality and foreshadow measures of wealth redistribution within cyberspace. To this end, we can think of ‘*digital-social rights’* as social rights belonging to the digital sphere and aimed at establishing redistribution policies among the citizens of cyberspace. More precisely, I defend the idea of the ‘digital-social’ right to fair compensation for producing data that generate gains for third parties and are used to design digital products and develop AI-based technologies―what has also been referred to as the ‘right to monetize’ (De Michelis & Bolognini 2018). This right connects research on digital rights to debates on basic income, as it makes it possible to think of a right to a ‘digital basic income’ (DBI) to be funded through the redistribution of data-generated revenues. The implementation of such a right would require designing a scheme for digital taxation and redistribution as an additional dimension of welfare policies. Just as 20th -century social citizenship has functioned as a ‘machine’ offsetting the imbalances produced by industrial technologies, this system of automated taxation and redistribution of data revenues should be designed to mitigate the new social inequalities generated by disruptive platform technologies.

The term ‘*cloud welfare*’ may tentatively be used to label this project for the redistribution of data-generated revenues based on the ‘digital-social right’ of internet users to receive a share of the income resulting from their online activities. It would consist in an automated system for the taxation of data-generated revenues and for their redistribution in the form of a digital currency that may have several applications. Like all major social rights, the digital-social right to a digital basic income related to data production and supply should be considered universal―i.e. simply related to the status of digital citizens―and not proportionate to the amount of data supplied by different individuals. A digital income tax will subsequently redistribute DBI to specific beneficiaries. Thus, such an automated system of digital redistribution would not result in a race toward digital clicks; rather, it could promote a shift toward less addictive digital services, as clicks would carry less monetary value for digital companies. It is important to note that this perspective is primarily designed for the EU data market, since it is based on the digital rights established by the growing EU digital regulations, especially the GDPR, and on their possible developments―e.g., through the forthcoming Digital Services, Digital Markets, and Data Governance Acts (Husson 2020), the ECB digital Euro project, and the EU proposal for a Web Tax.

Yet, the very opportuneness and feasibility of the form of ‘digital redistribution’ just outlined are far from self-evident and immediately raise three main concerns. First, a structural tension might soon emerge between digital privacy and redistribution measures based on data analysis―something I will consider in the conclusion. A second major obstacle pertains to the gap between the national scale inherent in traditional redistribution policies and the inherently supranational nature of cyberspace and data streaming―an issue which I will address separately in a forthcoming article. Lastly, the principles of digital-social citizenship I have outlined would make it necessary to enable digital users to dispose of their data under some kind of property scheme, which might be problematic since many legal scholars take the view that data cannot be owned (Hummel et al. 2020). This is because data are non-rivalrous: their possession does not exclude the use by third parties, and therefore it should be understood in terms of access rather than property (Purtova 2015). Furthermore, personal data often have no value in themselves; they are just raw material that has storage costs and requires complex and expensive infrastructures for the extraction and monetization of its value. The latter only emerges after aggregation and processing mechanisms through which data lose any personal and property features. It is precisely on this ground that the GDPR has revealed its main weaknesses, since it does not immediately encompass a broad range of value-generating activities concerning data that cannot be readily characterized as ‘personal.’ The purpose of this article is to outline a framework for ‘digital redistribution’ rather than describe its possible mechanisms of implementation, but it is clear that the latter would require a notion that extends beyond ‘personal’ data as defined under the current regulatory framework and overcomes the rigid distinction between personal and non-personal data to include several kinds of metadata. Such a notion should ultimately encompass ‘any data a given data subject is generating, through one or more devices, through one or more connections,’ and ‘all data which will be generated by IoT devices for the benefit of an enterprise’ (De Michelis & Bolognini 2018: 4). This points to the notions of ‘permission rights’ and ‘data reuse,’ and to the ‘purpose’ of the data collection that the GDPR has made compulsory to declare and receive consent to. Thus, privacy settings, together with geography and the time-length of the connection, could be used as a proxy for measuring the potential commercial value of users’ data, which should serve as the basis for designing schemes of digital redistribution.

## Conclusions

The contemporary data-driven digital economy fosters new inequalities in wealth and income distribution in the context of an enduring erosion of the social policies established in the past century. The COVID-19 crisis has accentuated this issue through the massive surge in demand for public, retail, and corporate digital services provided by Big Tech multinationals. This is consolidating the dominant market position of the latter, which are likely to emerge from the pandemic stronger than before (Billotta 2020). How Tech Giants should be taxed has thus become a pressing issue, especially in the European Union, which represents the biggest data market and a leader in tech regulation but remains non-competitive in the global digital economy―as highlighted by the French President’s caveat: ‘The US have the GAFA, China has the BATX. And Europe? We have the GDPR’ (Husson 2020). Thus, a debate has grown concerning a possible European ‘web’ or ‘tech’ tax based on a new system of tax residency for those big companies selling digital services and creating value from users’ data. Proposals on this matter have been advanced by the EU Commission (2018a, 2018b), which has even featured such a tax as a source of funding for the post-pandemic recovery plan. This debate has been held back to avoid conflict, especially with the US, in the OECD negotiations on a minimum corporate tax, but will surely regain relevance in the coming future. The purpose of this article has been to advance a rights-based approach to such a debate by exploring the historical interplays between technological changes and citizenship rights in order to design a new social rights regime for the digital era. This aim has led me to introduce the idea of ‘digital-social rights’ resulting from the incorporation of the social principles of welfare and redistribution into emerging digital rights. Hence, I have outlined the ways in which a form of ‘digital basic income’ (DBI) could be implemented in the digital sphere to redistribute part of the revenue generated by the users’ data in favor of producers and suppliers.

This prospect of ‘digital redistribution’ might appear utopian in many respects. Some major obstacles must be overcome in order to make it possible, starting from the fact that it should be based on a supranational system providing ‘digital citizens’―those who use the internet on a daily basis―with a unique digital identity and legal persona (Orgad & Baubock 2018). However, a preliminary and even more complex step would consist in a public conversation on the political principles and normative justifications that underlie the ideas of digital-social rights and digital basic income. As a general starting point, we might argue that, just as the ‘industrial social contract’ has offered a certain living standard in exchange for submission to the discipline of wage labor and employment, the establishment of ‘digital-social rights’ should be based on a new ‘digital-social contract’ that exchanges our digital privacy for a form of digital income. In order to access the latter, digital citizens should indeed consent to their data becoming information that can be used to generate economic value, improve digital technologies, and develop public services. As such, this horizon might appear ‘functional’ rather than ‘critical,’ since it leaves unquestioned the digital commodification and manipulation of our lives, which is stressed in contemporary debates on ‘surveillance’ capitalism that point to the need to regain control of our data and privacy. Conversely, the idea of a ‘digital-social right’ to DBI assumes the inevitable ‘public’ relevance that those data have in our digital present and for future developments of the digital environment. In this sense, the evolution of digital-social citizenship might imply a trade-off with the dimension of privacy. Whether this would embody a utopian or dystopian horizon is a question that remains open. The outcome will largely depend on the initiative of ‘digital citizens’―that is to say, on the presence or absence of *political struggles* within cyberspace, whereby digital users approach digital rights as a battleground, as occurred with previous waves of citizenship rights. In turn, this raises the issue that the tools to enact digital citizenship are currently owned by commercial platforms, which commodify digital acts and reduce digital citizens to users and customers with very little agency (Hintz et al. 2019). Accordingly, the analytical comparison between ‘industrial’ and ‘digital’ citizenship should further be developed to encompass―in addition to the dimension of rights that has been the focus of this article―the kinds of political agency which these forms of citizenship entail.

## Data Availability

Not applicable.

## Footnotes

1 Tocqueville 1994: 556. *How an Aristocracy May Be Created by Industry* is the title of Chap. 20 of *Democracy in America* (Vol. 2).

2 For Marx (1844) this asymmetry between the legal-political sphere and the social dimension was the defining feature of modern political revolutions, which established the modern ‘citizen’ as a merely formal figure bearing a generic right to equality that exists only in the civic and political dimensions, which are abstract, but not in the social sphere, where real life takes place and from which law and politics originate. Polanyi (2001: 267–8) described the rise of social welfare as the result of this 19th -century ‘discovery of society,’ which ‘came to us through living in an industrial society.’

3 The genealogy of these principles can be traced back to the English Factory Act of 1833 and the 1841 French law on child labor as pioneering attempts to reshape the industrial fabric with the aim of protecting a category of citizens through social security measures (Lynch 1988; Weissbach 1989). The German social legislation adopted in the 1880s then marked the first consistent codification of the welfare state, while the 1919 Weimar constitution inaugurated the constitutionalization of universalistic social rights (Ritter 1986).

4 ‘Trade unionism has, therefore, created a secondary system of industrial citizenship parallel and supplementary to the system of political citizenship’ (Marshall 1992: 26). Other authors subsequently used ‘industrial citizenship’ to describe the role of trade unions in public life and the forms of active citizenship in industrial workplaces (Streeck 1997; Bagguley 2013; Stranglemann 2015).

5 The preamble to the French Constitution of 1946 states that ‘Everyone has the duty to work and the right to obtain a job.’ The 1948 Italian Constitution grants ‘to all citizens the right to work’ and commits the State to promoting ‘the conditions that make this right effective’ (art. 4). The 1978 Spanish Constitution affirms ‘the duty to work and the right to work’ (art. 35). The 1974 Greek Constitution defines work as ‘a right’ and places it ‘under the protection of the State’ (art. 22).

6 In particular, financial capitalism––with the development of products and techniques such as derivatives and securitization––overlaps with technological changes in fracturing the industrial nexus between labor and citizenship: see Boyer (2000), Krippner (2012), Hager (2016), Lemoine & Ravelli (2017).

7 Milton Friedman (1962) proposed a scheme whereby all people below a certain income threshold would receive a public subsidy as a supplemental reimbursement from the government instead of paying taxes. Many UBI supporters argue that the NIT should not be considered a form of basic income but rather a model aimed at employed persons whose capacities are not enough to meet their own needs (Straubhaar 2017: 75; Zwolinski 2012).

8 The growing relevance of human capital, knowledge and information in the digital economy also highlights the key role of education and therefore the ‘right to long-life learning’ as another possible pillar of post-industrial welfare.

9 See statista.com or emarketer.com for details.

10 I have defined ‘industrial citizenship’ by highlighting the gender hierarchy ingrained in the division between paid ‘productive’ and unpaid ‘reproductive’ labor. Hence, it is important to also assess how gender and other biases can impact access to digital and ‘digital-social’ rights, and can be reproduced by algorithmic technologies (Ruha 2019; Kotliar 2020).
